# Epigenetic Alterations Upstream and Downstream of p53 Signaling in Colorectal Carcinoma

**DOI:** 10.3390/cancers13164072

**Published:** 2021-08-13

**Authors:** Maja T. Tomicic, Mona Dawood, Thomas Efferth

**Affiliations:** 1 Department of Toxicology, University Medical Center, 55131 Mainz, Germany; tomicic@uni-mainz.de; 2 Department of Pharmaceutical Biology, Institute of Pharmaceutical and Biomedical Sciences, Johannes Gutenberg University, 55128 Mainz, Germany; modawood@uni-mainz.de

**Keywords:** acetylation, carcinogenesis, methylation, micro-RNA, oncogene, signal transduction, tumor suppressor

## Abstract

**Simple Summary:**

Colorectal cancer (CRC) belongs to the most common cancer types. It is well known that half of all CRC possess missense mutations in the *TP53* tumor suppressor gene. However, the entire signaling cascade upstream and downstream of the p53 protein may also contribute to CRC development, if relevant players in this signaling cascade lost their function. Besides p53 loss-of-function by mutations, epigenetic changes (DNA methylation, post translational modifications of histones, micro-RNAs) play a vital role in CRC development. In the present review, we concentrated on the epigenetic modifications related to the entire p53 signal transduction cascade upstream and downstream of p53. Indeed, numerous epigenetic aberrations influence the tumor suppressor function of p53 independent of missense mutations. Thus, the role of p53 for CRC development, therapy response and survival prognosis of patients may be much more complex than predicted earlier. Hence, we are in need to use novel diagnostic methods that are capable of evaluating the genetic and epigenetic changes in the “p53 signalome”, so that diagnosis and management of CRC will improve.

**Abstract:**

Colorectal cancer (CRC) belongs to the most common tumor types, and half of all CRC harbor missense mutations in the *TP53* tumor suppressor gene. In addition to genetically caused loss of function of p53, epigenetic alterations (DNA methylation, histone modifications, micro-RNAs) contribute to CRC development. In this review, we focused on epigenetic alterations related to the entire p53 signaling pathway upstream and downstream of p53. Methylation of genes which activate p53 function has been reported, and methylation of *APC* and *MGMT* was associated with increased mutation rates of *TP53*. The micro-RNA 34a activates *TP53* and was methylated in CRC. Proteins that regulate TP53 DNA methylation, mutations, and acetylation of TP53-related histones were methylated in CRC. P53 regulates the activity of numerous downstream proteins. Even if *TP53* is not mutated, the function of wildtype p53 may be compromised if corresponding downstream genes are epigenetically inactivated. Thus, the role of p53 for CRC development, therapy response, and survival prognosis of patients may be much more eminent than previously estimated. Therefore, we propose that novel diagnostic devices measuring the entirety of genetic and epigenetic changes in the “p53 signalome” have the potential to improve the predictive and prognostic power in CRC diagnostics and management.

## 1. Introduction

Since the early 19th century and the ground-breaking experiments of Gregor Mendel, genetics is fundamental in biology in general and also specifically in cancer biology [1]. Epigenetics emerged as new field, which complements many genetic mechanisms in evolution of life on earth, organismic homeostasis, and pathophysiology of diseases as well. Epigenetics focuses on changes in gene function, which are not caused by changes in DNA (mutation, recombination), but which are inherited from one cell to another. Epigenetic changes are related to the methylation of DNA or RNA, the modifications of chromatin and the expression of micro-RNAs.

DNA methylation is catalyzed by DNA-methyltransferases (DNMTs). DNMT1 maintains the methylation pattern of DNA, while DNMT3a and DNMT3b are responsible for de novo methylation. RNA methylation is catalyzed by DNMT2 [2,3]. There is a balance between methylation and demethylation of nucleic acids. Demethylation reactions are catalyzed by DNA methylase. DNMTs preferentially methylate cytosine within CpG islands. CpG islands are CG-rich DNA regions upstream of genes, which comprise gene promoters and regulate gene expression by different degrees of methylation. It has been estimated that about one half of all CpG islands is located in housekeeping genes and that about 40% of all gene promoters contain CpG islands [4,5]. CpG islands are domains for the recruitment of RNA polymerase II and transcription factors to initiate the transcription process [6]. CpG methylation favors the binding of methyl-binding proteins, which results in a nucleosome condensation and thereby inhibition of transcription. 

DNA is bound to histones (histone 2A, histone 2B, histone 3, and histone 4), which together form the nucleosome. Histones can be methylated, acetylated, and/or phosphorylated at lysine, histidine and arginine residues. The acetylation of histones by histone acetyltransferases (HATs) opens the nucleosome conformation and thereby enables transcription by RNA polymerase II. The closure of open chromatin leading to transcriptional repression is conferred by methyl-binding proteins [7]. 

In contrast to HATs, histone deacetylases (HDACs) remove the acetyl group from the lysine residues and restore the interaction between DNA and histones. As a result, the histone-DNA architecture is stabilized leading to the inhibition of transcription process [8]. Similar to yeast deacetylases, HDACs are classified into four major types: class I HDACs (HDAC1-3, and HDAC8), class II HDACs (HDAC4-7, HDAC9, and HDAC10), class III HDACs (also named sirtuins) (SIRT1-7), and class IV which consists only of HDAC11 [9].

Micro-RNAs (miRNAs) are short, about 22 nucleotide long, non-coding RNA sequences, which are complementary to messenger RNA (mRNA) sequences [10]. Because of their complementarity, they can bind to corresponding mRNA species. Double-stranded miRNA-mRNA molecules are cleaved by RNases [11,12]. Thereby, miRNAs can silence gene expression. 

Taken together, gene expression in mammals is epigenetically regulated by DNA methylation, histone acetylation, and micro-RNAs. As the basic scaffold of the DNA is neither changed by DNA-methylation, nor by histone acetylation and miRNAs, these modifications are not referred to as genetic mutations.

During the past years, it became evident that epigenetics plays an eminent role for carcinogenesis, because the silencing of tumor suppressor genes can happen both by classical genetic mechanisms (e.g., point mutations, chromosomal aberrations) as well as epigenetic modifications [13]. It is widely accepted that carcinogenesis evolves stepwise: (1) initiation with DNA mutation, (2) promotion with clonal expansion and proliferation of initiated cells, and (3) progression with chromosomal instability, neoangiogenesis, and metastasis [14,15]. Epigenetic changes can occur in all three stages of cancer development [13].

A central player in carcinogenesis is the tumor suppressor gene *TP53*. Its encoded protein p53 acts as transcription factor, which arrests the cell cycle after DNA damage and either contributes to DNA repair or triggers the induction of apoptosis [16,17]. Besides, p53 plays a prominent role in the regulation of cellular senescence and organismal aging [18,19,20]. Mutations cause a loss of function and are critical for the development of cancer, since damaged cells continue to divide and become malignant [21]. Remarkably, 50–60% of all colorectal cancers harbor mutations in the *TP53* gene, most of which are missense mutations at certain hot spots of the gene [22]. 

In general, mutations can be categorized as driver mutations, which provide a survival advantage to tumor cells, and passenger mutations. In addition to mutations in *TP53*, other driver mutations in colorectal cancer (CRC) are found in genes of the WNT signaling pathway (e.g., *APC* and *CTNNB1*), *TGF-β*, *DCC*, *SMAD*, *KRAS*, *RAF*, *BRAF*, *PI3K*, *PTEN* as well as in the DNA mismatch repair genes *hMSH3* and *hMSH6,* beyond other mutations [23]. 

Mutations in DNA mismatch repair genes do not only predispose to mutations in driver genes, but also to another characteristic feature of colorectal cancer, i.e., microsatellite instability (MSI) [24]. Because of impaired DNA mismatch repair, there is a predisposition for DNA mutations, and three phenotypes can be distinguished: MSI stable, MSI low, and MSI high, the latter one with a better prognosis than the others [25]. Epigenetic inactivation of other DNA repair genes contributes to mutations of driver genes in CRC (e.g., the mismatch repair genes *hMLH1*, *hMSH2*, and the gene coding for *O*^6^-methyguanine-DNA methyltransferase, *MGMT*) [26].

Interestingly, epigenetic alterations occur more frequently in colorectal cancer than gene mutations. A large number of hyper- and hypomethylations appear in genes and miRNAs of a majority of colorectal cancers [27]. It is estimated that 600–800 genes are transcriptionally silenced by CpG island methylation and that miRNAs also considerably affect transcriptional repression in this tumor entity [28,29]. The list of genes belonging to this CpG island hypermethylated phenotype (CIMP) is still growing and this phenotype is of clinical prognostic significance [30,31,32]. Several different epigenotypes have been defined in colorectal cancer with different prognostic outcome (high and low methylator phenotypes associated with specific high and low MSI and mutational profiles in driver genes [33,34]).

Even if CIMP epigenotypes can be identified consisting of different genetic and epigenetic profiles, there are numerous specific interactions between single genes (e.g., *TP53*) and aberrant methylation in p53-related genes. 

A lot of attention has been paid on mutations in the *TP53* gene and the related loss of function. Rather than genetic alterations, we focus in the present paper on epigenetic changes in the *TP53* gene and also on epigenetics in the signaling cascade upstream and downstream of the p53 protein. If we assume that epigenetic changes in genes upstream and downstream of p53 also contribute to alterations in p53 signaling, the oncogenic potential of the entire epigenetically silenced p53 signaling pathways in addition to 50–60% mutations in *TP53* itself is much larger than estimated yet. Therefore, we performed a systematic review on the interactions between p53 and methylation changes in genes upstream of the p53 signaling cascade or downstream genes, which are regulated by p53. Our overview is based on a PubMed search with the keywords “colorectal + epigenetic + p53” and “colorectal + methylator phenotype + p53” as of 19 May 2021.

## 2. Epigenetic Alterations Upstream of p53

### 2.1. Methylation Status of p53-Activating Genes

**Figure 1:**
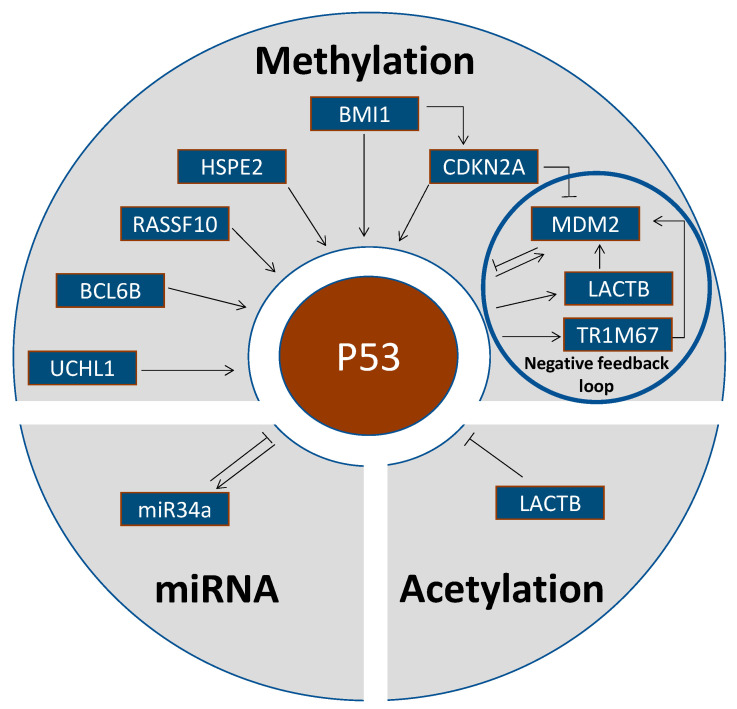
Epigenetic alterations upstream of p53.

A number of genes mostly acting as tumor suppressors or oncogenes regulate the activity of p53 (Table 1, Figure 1).

Ubiquitin carboxyl-terminal hydrolase L1 (UCHL1) is a tumor suppressor protein, which stabilizes p53 expression. Its demethylation causes growth inhibition by G2/M cell cycle arrest and apoptosis induction. *UCHL1* methylation has been reported in 22/31 CRC cases (=71%) [35].

B-Cell CLL/lymphoma 6 member B (BCL6B) is a tumor suppressor protein, which activates p53 signaling and induces apoptosis. The *BCL6B* gene was methylated in 81/102 CRC cases (=79%) [36]. 

Ras-association domain family member 10 (RASSF10) is a RAS-associated domain family member, which activates p53 signaling and sensitizes to anti-cancer drugs (e.g., docetaxel). Demethylation of *RASSF10* induced apoptosis and inhibited proliferation in 54/89 CRC cases (=61%). The *RASSF10* methylation status was positively associated with tumor stage and metastasis [37]. 

Heparanase 2 (HPSE2) acts as tumor suppressor, which regulates p53 signaling leading to G1 arrest of the cell cycle. Hypermethylation of *HPSE2* significantly correlated with shorter survival times of CRC patients [38]. 

The proto-oncogene *BMI1* (B lymphoma Mo-MLV insertion region 1 homologue) is an epigenetic repressor by remodeling chromatin. P14^ARF^ and wildtype p53 were upregulated in *BMI1*-mutated CRC cases [39]. P14^ARF^ is required for the induction of p53 expression and apoptosis.

The protein stability of p53 is regulated by p14^ARF^, which is also designated as cyclin-dependent kinase inhibitor 2A (CDKN2A). This protein interacts with MDM2 (mouse double minute 2 homologue) and thereby antagonizes the MDM2-dependent degradation of p53. Demethylation of the *CDKN2A* promoter was associated with MDM2 expression, while *CDKN2A* hypermethylation resulted in cytosolic translocation and inactivation of MDM2. Thereby, *CDKN2A* hypermethylation abrogated the activity of wildtype p53. *CDKN2A* hypermethylation was more frequently found in tumors with wildtype p53 than in those with mutated p53 [40]. *CDKN2A* demethylation was associated with nuclear (i.e., active) MDM2 expression in CRC [41]. In CRC, *CDKN2A* promoter hypermethylation was significantly correlated with p53 overexpression and MDM2 overexpression [42].

### 2.2. Methylation Status of p53-Inhibiting Genes

In addition to genes that activate p53, epigenetic regulation of genes, whose gene products inhibit p53, has also been reported (Table 1). 

The tumor suppressor APC (adenomatous polyposis coli protein) inhibits β-catenin, and β-catenin overexpression increases the *TP53* mutation rate. Hypermethylation of APC was associated with p53 mutation in 208 CRC cases [43,44]. 

O^6^-Methylguanine-DNA methyltransferase (MGMT) is a DNA repair protein whose downregulation by promoter hypermethylation predisposes genes to p53 mutations. *MGMT* promoter hypermethylation was significantly correlated to G > A transition mutations in the *TP53* gene in 314 CRC patient samples [45]. This result was confirmed by other authors [46]. However, there was no association between *MGMT* methylation and *TP53* mutations in 261 CRC biopsies from Afro-American patients, indicating that population-based differences may exist [47].

Deficiency of the tumor suppressor pentraxin 3 (PTX3) increases susceptibility to carcinogenesis by increasing DNA damage, p53 mutations and inactivation of p53 downstream signaling (MDM2, BAX, and CDKN1A/p21). Increasing rates of *PTX3* promoter methylation were observed from normal colon epithelium to adenomas and CRC biopsies [48].

Trimethylated histone H3K27 binding in the *PCAF* (P300/CBP-associated factor) promoter attenuated transcription of this gene. Decreased PCAF impaired the acetylation of p53 and attenuated the p53-dependent transcription of p21, which resulted in increased cyclin D1 expression and Retinoblastoma 1 (RB1) phosphorylation as well as increased resistance to 5-fluorouracil [49].

Lactamase β (LACTB) is a tumor suppressor, which binds to the C-terminus of p53 to inhibit MDM2-mediated p53 degradation. LACTB was significantly downregulated in CRC due to promoter methylation [50]. 

TRIM67 (Tripartite motif containing 67) is also a tumor suppressor. Under stress conditions, p53 binds to the TRIM67 promoter and upregulates TRIM67 expression in a TRIM67/p53 self-amplifying loop. TRIM67 interacts with the C-terminus of p53 to inhibit MDM2-mediated p53 degradation. In 108/138 CRC (=79%), TRIM67 expression was downregulated due to promoter methylation. Demethylation by treatment with 5-aza-2′-deoxycytidine restored TRIM67 expression in CRC cells [51].

### 2.3. Methylation Status of Other Genes

Epigenetic alterations have been associated with p53 without explicitly describing the direct causative relationship to p53 function (Table 1 and Table 2).

Cyclooxygenase 2 (COX2) is a proinflammatory enzyme, which is involved in the progression of CRC. Methylation of the *COX2* gene was found in 12/93 CRC biopsies (=13%) and 7/50 colorectal adenomas (=14%). *COX2* methylation was inversely related to *TP53* mutations, albeit the functional relevance of these mutations is not clear yet [52].

A CpG island methylator phenotype (CIMP) can be found in a fraction of CRC. CIMP was discussed as a predisposing factor for CRC carcinogenesis. *TP53* mutations have been found in 10/41 CIMP-positive CRC (=24%) compared to 30/46 CIMP-negative cases (=60%) [53]. Another study also reported that CIMP correlated with wildtype p53 [54].

### 2.4. Acetylation Status of p53-Inhibiting Genes 

LACTB was not only downregulated in CRC due to promoter methylation, but also due to histone deacetylation [50] **(**Table 1 and Table 2).

### 2.5. P53 Regulation by Micro-RNAs

Micro-RNAs are involved in the regulation of p53 and its network at multiple levels [57] (Table 1 and Table 2). This can occur by direct p53 targeting or indirectly by targeting p53 regulators (e.g., MDM2 and MDM4). Vice versa, p53 is a transcriptional regulator of numerous miRNAs, which contributes to its tumor suppressive function. 

In tumor cells, including CRC, miRNAs can function in a tumor-suppressive (protective) or tumor-promoting (oncogenic) manner. MiR-339-5p was frequently downregulated and associated with poor patients’ prognosis; miR-339-5p and miR1827 directly repressed MDM2 expression through binding to *MDM2* 3′-UTR, which elevated p53 protein expression and p53-mediated apoptosis and senescence. In parallel, it also inhibited migration, invasion, and the growth of CRC xenografts [58]. Other tumor-suppressive miRNAs targeting the MDM2/p53 axis were miR193a-5p and miR-146a-5p [59]. MiR-1249 is a direct transcriptional target of p53, and p53-mediated induction of miR-1249 inhibited tumor growth, metastasis, and angiogenesis in vitro and in vivo [60]. Ectopic expression of miR-133a markedly increased p53 levels and induced p21 transcription and, thus, significantly suppressed CRC cell growth in vitro and in vivo, and sensitized cells to doxorubicin and oxaliplatin [61]. The p53-induced miR-34 microRNA family mediated repression of c-Kit by p53 via a conserved seed-matching sequence in the *cKIT* 3′-UTR; ectopic c-Kit expression conferred resistance of CRC cells to 5-FU, whereas ectopic miR-34a sensitized the cells to the drug [62]. MiR-34a directly inhibited the oncogenic receptor tyrosine kinase CSF1R, and p53 repressed CSF1R by inducing miR-34a. Accordingly, resistance of CRC cells to 5-FU was mediated by miRNA-34a silencing (via CpG-methylation) and the resulting elevated expression of CSF1R [63]. In CRC cells, the transcription factor AP4 was downregulated by p53, which was indirectly mediated by the tumor-suppressive miR-15a and miR-16-1, targeting the 3′-UTR of *AP4* mRNA, inducing mesenchymal-epithelial transition (MET) and inhibiting CRC cell migration and invasion [64]. MiR-16 repressed CRC cell growth by decreasing Survivin (BIRC5) expression through a direct targeting of *BIRC5*, and p53 negatively modulated BCL-2 by controlling miR-1915 [65,66]. Also, miR-148b, whose transcription is directly activated by p53, bound specifically to the 3′-UTR of *P55PIK* mRNA and suppressed p55PIK expression, which abolished proliferation and cell cycle progression of CRC cells and decreased tumor growth in vivo [67]. Furthermore, miR-143 and miR-145 function in a tumor-suppressive way, and the major mediators of the oncosuppression were genes belonging to the growth factor receptor-mitogen-activated protein kinase network and to the p53 signaling pathway [68]. In rectal tumors, elevated expression of miR-150-5p and miR-196b-5p significantly increased patients’ survival [69]. In a genome-wide systematic approach, miR-30e, a direct transcriptional target of p53, was the most frequently deregulated miRNA in a p53-deficient background of CRC [70]. MiR-600 represents a direct negative regulator of p53 through binding the 3′ UTR of the *TP53* mRNA. Its overexpression decreased endogenous levels of mutant p53 and inhibited cell proliferation, migration, and invasion in mutant p53-expressing CRC cells [71]. Furthermore, the WNT/cMYC axis signaling inhibited the expression of p53 by promoting a direct targeting of p53 by miR-552 leading to resistance to drug-induced apoptosis, suggesting that miR-552 may function as an oncogene [72]. Furthermore, miR-27a was also identified to be oncogenic in CRC cells. The overexpression of miR-27a, i.e., its binding to two putative binding sites on the 3′-UTR of the *TP53* mRNA, resulted in the decreased p53 expression [73]. MiR-300 was a direct positive regulator of p53 through binding to the 3′UTR of *TP53* in mutant p53 CRC cells. Both miR-300 and p53 induced EMT, thus being oncogenic [74]. One more CRC-promoting miRNA described so far is miR-150-5p repressing the p53 pathway [75].

Micro-RNA 34a acts as translational regulator, which activates p53 by inhibiting its acetylation by *MTA2* (Metastasis Associated 1 Family Member 2) and HDAC1 [55]. The methylation of miR34a correlated with the p53 wildtype status in CRC and other tumor types [56].

**Table 2:** In vitro and in vivo studies on epigenetics of p53 in CRC.

Gene Name	Epigenetic Event	Cellular Function	Reference
Lactamase β (LACTB)	*LACTB* promoter methylation and histone deacetylation	Activation of CRC cell proliferation, migration, and invasion in HCT116 and HCT8 cells. Inhibited CRC xenograft growth and metastasis in vivo	Zeng et al., 2018 [50]
Deregulation of P53	Abnormalities of miRNA expression	p53 effects (e.g., cell cycle arrest and apoptosis) were phenocopied in HCT-116 cells	Nugent et al., 2011 [76]
O6-methylguanine DNA methyltransferase	Aberrant methylation of *APC, MGMT*, *hMLH1*, *P16*, *N33*	High levels of microsatellite instability in 208 CRC patients	Suehiro et al., 2008 [43]
Inhibitor of growth 2 (ING2)	ING2 is a cofactor of p300 for p53 acetylation	Positive regulation of p53-mediated replicative senescence in young fibroblasts	Pedeux, et al., 2020 [77]
NAD^+^-dependent protein deacetylase SIRT1	Deacetylation of p53	Decrease of cell proliferation and induction of apoptosis in vivo and in CRC patients	Stünkel et al., 2007 [78]

## 3. Epigenetic Alterations at p53 as Target Site

### 3.1. Methylation of the TP53 Gene 

The TP53 gene is a direct target site for epigenetic regulation (Table 3). The methylation inside and outside of exonic CG sequences of the *TP53* gene was correlated with point mutations. Cigarette smoking increased the occurrence of methylation-associated mutations [79]. Methylation of the *TP53* gene, histone modifications, chromatin remodeling, and non-coding RNAs were significantly associated with colitis-related carcinogenesis and tumor progression [80].

Polymerase epsilon (POLE) is a replicative polymerase important for efficient replisome assembly and strand synthesis, supporting tumor suppression [81]. POLE is involved in DNA repair and especially methylated cytosines (5mCs) are frequently mutated. Mutations in POLE exonuclease domain increase 5mC mutagenesis and a mutator phenotype. CRC with mutated POLE frequently contain highly methylated CpG islands in the *TP53* gene [82].

PHD finger protein 2 (PHF2) is a tumor-suppressing histone demethylase. It demethylates the repressive H3K9-Me2 mark in chromatin to induce p53 transcription. PHF2 was downregulated in CRC and PHF2 correlated with p21 in cancers expressing functional p53 [83]. 

Histone lysine-specific demethylases 1 and 2 (LSD1, LSD2) are demethylases that bind to p53 and demethylate K370me2 at Lys370 (LSD1) or K3K4me2 in the *TP53* promoter (LSD2). This leads to the inhibition of p53 function and p53-mediated apoptosis [83,84,85,86].

### 3.2. Acetylation of p53

In addition to methylation, epigenetic regulation of p53 can also take place by acetylation (Table 3). NRDC is a histone-binding protein that binds HDAC1 and inhibits HDAC1 recruitment to the *TP53* promoter and p53 acetylation [87,88].

**Table 3:** Epigenetic alterations at *TP53* as target site.

Gene	Gene Name	Gene Function	Epigenetic Event	Reference
*TP53*	Tumor suppressor 53	Tumor suppressor	Methylation inside and outside of exonic CG sequences was correlated with point mutations. Cigarette smoking increased the occurrence of methylation-associated mutations.	Kouidou et al., 2006 [79]
*TP53*			DNA methylation, histone modifications, chromatin remodeling, and non-coding RNAs were significantly associated with colitis-related carcinogenesis and tumor progression.	Saraggi et al., 2017 [80]
*POLE*	Polymerase ε	Involved in DNA repair and chromosomal DNA replication. Methylated cytosines (5mCs) are frequently mutated. POLE exonuclease domain mutations increase 5mC mutagenesis and a mutator phenotype.	Highly methylated CpGs in *TP53* contain mutation hotspots in POLE-mutated CRC	Poulos et al., 2017 [82]
*PHF2*	PHD finger protein 2	Tumor suppressor. Histone demethylase. PHF2 demethylates the repressive H3K9-Me2 mark in chromatin to induce p53 transcription.	PHF2 was downregulated in CRC and PHF2 correlated with p21 in cancers expressing functional p53.	Lee et al., 2015 [83]
*LSD1*			LSD1demethylated K370me2 (dimethylated Lys370) of p53, which cannot bind to DNA and is inactivated. LSD1 inhibits p53-mediated apoptosis.	Huang et al., 2007 [84]
*LSD1*	Histone lysine-specific demethylase 1	Lysine-specific demethylases that removes histone methylations catalyzed by histone methyltransferases.	LSD1-knockout in HCT116 cells did not increase H3K4me2 (dimethyl-H3K4) or change stability or function of p53.	Jin et al., 2013 [85]
*LSD2/KDM1B*	Lysine-specific histone demethylase 1B	Oncogene. LSD2 directly binds to *TP53* and represses p53 expression via H3K4me2 demethylation at the *TP53* promoter.	LSD2 bound and inhibited p53 activity in CRC.	Cai et al., 2020 [86]
*NRDC*	Nardilysin, N-arginine dibasic convertase	NRDC is a dimethyl-H3K4 binding protein.	Nuclear NRDC bound to histone deacetylase 1 (HDAC1) and inhibited HDAC1 recruitment to the *TP53* promoter and p53 acetylation.	Li et al., 2012; Kanda et al., 2018 [87,88]

## 4. Epigenetic Alterations Downstream of p53

### 4.1. Methylation Status of p53 Downstream Genes 

**Figure 2:**
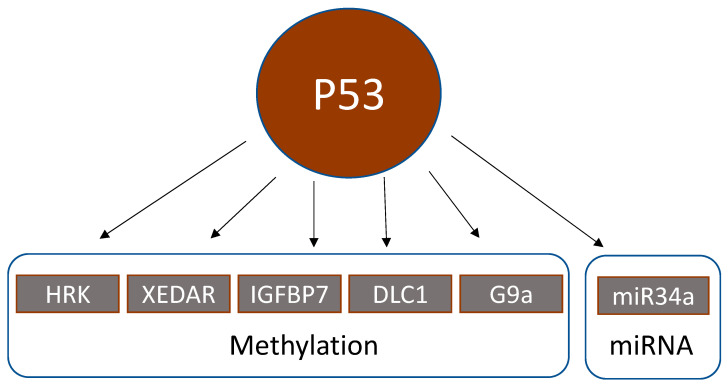
Epigenetic alterations downstream of p53.

Genes that are activated by p53 still can lead to inactive downstream signaling, if they are methylated (Table 4, Figure 2).

The BCL-2-interacting protein HRK interacts with BCL-2 and is a pro-apoptotic p53 target protein. Its expression can be inhibited by the methylation inhibitor 5-aza-2′-deoxycytidine, an effect which is further enhanced by histone deacetylase inhibitors (trichostatin A, depsipeptide). The methylation of the *HRK* promoter significantly correlated with the p53 wildtype status in 58 CRC cases [89].

The X-linked ectodermal dysplasia receptor (XEDAR) is a member of the tumor necrosis factor receptor family. P53 upregulates XEDAR expression through two p53-binding sites within intron 1 of the *XEDAR* gene. Inactivation of *XEDAR* results in enhanced cell adhesion and spreading, and resistance to p53-induced apoptosis. The expression of XEDAR was down-regulated by promoter hypermethylation or *TP53* mutations in CRC cell lines and clinical biopsies [90]. 

The expression of the insulin-like growth factor-binding protein 7 (IGFBP7) is induced by binding of p53 to a p53-responsive element in intron 7 of the *IGFBP7* gene. *IGFBP7* methylation was significantly higher in 83 CRC biopsies than in normal colonic tissue. 5-Aza-2′-deoxycytidine restored p53-induced IGFBP7 expression. *IGFBP7* methylation significantly correlated with wildtype p53 [91].

DLC1 (deleted in liver cancer 1) is a tumor suppressor. The DLC1-i4 is an isoform whose promoter is activated by wildtype p53. *DLC1-i4* was methylated in 2/4 CRC cell lines (=50%), and demethylation by 5-aza-2′-deoxycytidine restored its expression [92].

G9a (lysine methyltransferase 1C, euchromatic histone methyltransferase 2) is a histone methyltransferase, which demethylates the p53 protein at lysine 373. This reaction transactivated the expression of polo-like kinase 1 (PLK1) leading to increased proliferation [93].

### 4.2. Regulation of p53 by Acetylation

Several excellent reviews considered the various aspects of p53 acetylation. The acetylation of p53 at lysine 320 is achieved by histone acetyltransferase, PCAF (p300-CBP associated factor), which in most cases led to transactivation of the *P21* (*CIP1/WAF1/CDKN1A*) promoter and increased histone acetylation after DNA damage [94,95]. Moreover, the addition of an acetyl group to p53 protein increased its stability, its binding to low affinity promoters, and other proteins [96]. On the other hand, inhibition of histone deacetylases (HDACs) resulted in increased p53 acetylation and eventually p53-dependent activation of apoptosis, cell cycle arrest, and senescence [97]. 

### 4.3. Regulation of Micro-RNAs by p53

Although not investigated to great detail, p53 can regulate micro-RNAs, which represents another level of gene regulation downstream of p53 (Table 4). P53 activated the transcription of miR-34 family members. The miR-34b/c CpG island is a bidirectional promoter, regulating the expression of both miR-34b/c and B-cell translocation gene 4 (*BTG4*). Methylation of this promoter silenced *BTG4* transcription. The methylation of the miR34b/c CpG island was found in 100/111 CRC (=90%) [98,99]. 

**Table 4:** Epigenetic alterations downstream of p53.

Gene	Gene Name	Gene Function	Epigenetic Event	Reference
*HRK*	BCL-2-interacting protein	Proapoptotic p53 target protein, whose expression is reversible by methylation inhibitors (5-aza-deoxycytidine) and further enhanced by histone deacetylase inhibitors (trichostatin A, depsipeptide)	*HRK* promoter methylation significantly correlated with wildtype p53 in 58 CRC.	Obata et al., 2003 [89]
*XEDAR*	X-linked ectodermal dysplasia receptor	XEDAR is a member of the tumor necrosis factor receptor family. P53 upregulates XEDAR expression through two p53-binding sites within intron 1 of the *XEDAR* gene. Inactivation of XEDAR results in enhanced cell adhesion and spreading, and resistance to p53-induced apoptosis.	*XEDAR* downregulation by hypermethylation or *TP53* mutations in CRC cell lines and biopsies	Tanikawa et al., 2009 [90]
*IGFBP7*	Insulin-like growth factor-binding protein 7	P53 induces expression of IGFBP7 upon binding to a p53 response element in intron 1 of IGFBP7.	*IGFBP7* methylation was significantly higher in 83 CRC biopsies than in normal colonic tissue. 5-Aza-2′-deoxycytidine restored p53-induced IGFBP7 expression. *IGFBP7* methylation significantly correlated with wildtype p53.	Suzuki et al., 2010 [91]
*DLC1*	Deleted in liver cancer 1	Tumor suppressor. The functional *DLC1-i4* promoter is activated by wild-type p53.	DLC1 isoform 4 (*DLC1-i4*) was methylated in 2/4 CRC cell lines (=50%). Demethylation with 5-aza-2′-deoxycytidine restored DLC-i4 expression.	Low et al., 2011 [92]
*G9a/KMT1C/EHMT2*	Lysine methyltransferase 1C, euchromatic histone methyltransferase 2	Histone methyltransferase that catalyzes mono- and dimethylation of histone H3K9	G9a dimethylated p53 at lysine 373. This transactivated PLK1 expression and increased proliferation	Zhang et al., 2018 [93]
miR-34b/c	Micro-RNA 34b/c	p53 activates the transcription of miR-34 family members. The miR-34b/c CpG island is a bidirectional promoter, regulating the expression of both miR-34b/c and B-cell translocation gene 4 (*BTG4*). Methylation of this promoter silenced *BTG4* transcription.	miR-34b/c CpG island methylation in 100/111 (=90%) CRC	Toyota et al., 2008; Navarro and Lieberman, 2015 [98,99]

## 5. Epigenetic Alterations and Clinical Outcome upon Standard Chemotherapy of CRC

In the past 10 years, a huge amount of data on epigenetic alterations in CRC has been published. Interest was placed in particular on putative clinical implications, emerging prognostic and diagnostic biomarkers, as well as on the implementation of the gained knowledge in precision (personalized) oncology [100,101,102,103,104,105]. 

Here, we focus on the impact of the CpG island methylator phenotype (CIMP), the most frequent epigenetically altered biomarker in CRC, on clinical outcome (disease progression, metastasis, and survival) of patients having received standard chemotherapy with 5-FU, oxaliplatin, and/or irinotecan. In a randomized controlled trial, significant differences in overall survival were not observed between patients with CIMP (+) vs. CIMP (−) tumors after adjuvant administration of oxaliplatin [106]. Thus, CIMP did not seem to be a prognostic biomarker in oxaliplatin-treated patients with resected CRC. On the other hand, in another clinical trial, CIMP was of prognostic value for stage III CRC patients treated with adjuvant oxaliplatin. A large cohort of well-defined patients with stage III CRC, CIMP (+) phenotype was associated with a shorter overall survival and a shorter survival after recurrence [107]. This was verified by another study, in which the CIMP (+) phenotype was an adverse prognostic marker in patients with metastatic disease treated with 5-FU/oxaliplatin and 5-FU/irinotecan [108]. Interestingly, in another clinical trial patients with stage III, CIMP-positive, MMR-intact CRC exhibited longer survival times, if irinotecan was added to combination therapy with 5-FU and leucovorin; i.e., those patients benefited most from the FOLFIRI protocol [109]. Furthermore, hypermethylation of p16 was predictive of clinical outcome in metastatic CRC patients treated with cetuximab and FOLFIRI, irrespective of KRAS mutation [110]. The progression-free survival of metastatic CRC patients with CIMP (+) tumors, who received sequential therapy with 5-FU/oxaliplatin (FOLFOX) as the first-line treatment, followed by irinotecan-based second-line treatment, was inferior to that of patients receiving the reverse sequence. It was concluded that the high mutation burden in EGFR-related genes in CIMP (+) tumors may cause a lower response to anti-EGFR antibody (cetuximab) therapy [111]. Recently, epigenetically regulated gene expression profiles revealed four molecular subtypes with prognostic and therapeutic implications in CRC [112]. A recent paper described a novel epigenetic signature of 8 hypermethylated genes that were able to identify metastatic CRC individuals with poor prognosis to oxaliplatin and irinotecan, characterized by CIMP (+) and MSI-like phenotype. The expression of the 8-genes signature and MSI-enriching genes was confirmed in oxaliplatin- and irinotecan-resistant CRC cell lines [113].

## 6. Conclusions and Perspective

While the significance of missense mutations in the *TP53* gene has been recognized for a long time, the significance of epigenetic changes has only become clearer during the past few years. More and more data on epigenetic changes are being found not only in the TP53 gene itself, but also in genes upstream or downstream in the p53 signal transduction chain. Until now, it was assumed that about half of all CRC are affected by loss-of-function mutations in p53. If epigenetic changes upstream and downstream also have to be considered to contribute to CRC carcinogenesis, the significance of the entire p53 signaling pathway becomes much greater than previously assumed. 

This is not only of prognostic relevance for the survival time of CRC patients, but also plays a prominent role for the response to therapy, since p53 is not only a factor that promotes carcinogenesis and tumor progression, but also determines the success of chemotherapy and radiotherapy of tumors [114]. 

Many studies showed the importance of epigenetics of *TP53* in cancer in general. The methylation of *TP53* and p53-related genes were well studied in comparison to acetylation and micro-RNAs. Therefore, more research is required to better understand how the epigenetics of p53 contributes to CRC carcinogenesis. 

The entirety of all partners involved in signaling is referred to as the “signalome”. Therefore, we propose that the genetic and epigenetic characterization of the “p53 signalome” by the development of novel diagnostic devices based on biochips, DNA/RNA-sequencing, and other advanced technologies have the potential to significantly improve today’s diagnostic power in CRC management. For example, bisulfite treatment of genomic DNA in combination with high-throughput DNA sequencing enables the study of the genomic DNA methylation in general, and specifically, p53-dependent signaling pathway genes. This gold-standard (bisulfite-conversion) method has been adapted to fit with next-generation sequencing technologies and shotgun-sequencing approaches. Concerning the acetylation profile, the chromatin immunoprecipitation (ChIP) with high-throughput sequencing or DNA microarrays (ChIP-chip) is widely used.

## Figures and Tables

**Table 1:** Epigenetic alterations upstream of p53.

Gene	Gene Name	Gene Function	Epigenetic Event	Reference
*UCHL1*	ubiquitin carboxyl-terminal hydrolase L1	Tumor suppressor. Carboxyl-terminal ubiquitin hydrolase regulating cellular ubiquitin levels	*UCHL1* methylation in 22/31 CRC (=71%). UCHL1 binds to and stabilizes p53 by the ubiquitination pathway, *UCHL1* demethylation caused growth inhibition, G2/M arrest and induction of apoptosis.	Yu et al., 2008 [35]
*BCL6B*	B-Cell CLL/Lymphoma 6 Member B	Tumor suppressor. BCL6B activates p53 signaling and induces apoptosis.	*BCL6B* was methylated in 81/102 CRC (=79%).	Hu et al., 2015 [36]
*RASSF10*	Ras-association domain family member 10	RASSF10 activates p53 signaling and sensitizes to docetaxel. *RASSF10* demethylation induced apoptosis and inhibited proliferation.	*RASSF10* was methylated in 54/89 CRC (=61%) and was positively associated with tumor stage and metastasis.	Jin et al., 2015 [37]
*HPSE2*	Heparanase 2	Tumor suppressor. HPSE2 regulates p53 signaling and G1 cell cycle arrest.	*HPSE2* hypermethylation correlated with shorter survival times of CRC patients.	Zhang et al., 2021 [38]
*BMI1*	B Lymphoma Mo-MLV insertion region 1 homologue	Proto-oncogene. BMI1 is an epigenetic repressor by chromatin remodeling. p14ARF is silenced by BMI1.	p14ARF and wild-type p53 were upregulated in BMI1-mutant CRC. p14ARF is required for the induction of p53 and apoptosis.	Maynard et al., 2014 [39]
*CDKN2A*	Cyclin-dependent kinase inhibitor 2A	CDKN2A/p14ARF antagonizes MDM2-dependent p53 degradation.	p14ARF hypermethylation was increased in tumors with wildtype compared to mutated p53 without statistical significance.	Esteller et al., 2000 [40]
		p14ARF promoter demethylation was associated with nuclear MDM2 expression (active state), p14ARF promoter hypermethylation with cytosolic MDM2 expression (inactive state).	Nuclear MDM2 expression was associated with p14ARF promoter demethylation in 33 CRC.	Esteller et al., 2001b [41]
		Tumor suppressor. Regulates p53 protein stability by interaction with MDM2. p14ARF promoter hypermethylation abrogates wild-type p53 activity.	*CDKN2A* promoter hypermethylation was significantly correlated with restricted p53 overexpression and MDM2 overexpression. Epigenetic silencing of *CDKN2A* is a deregulating mechanism of the p53-MDM2-p14ARF pathway in CRC exhibiting restricted p53 overexpression.	Nyiraneza et al., 2012 [42]
*APC*	Adenomatous Polyposis Coli Protein	APC inhibits β-catenin, and β-catenin overexpression increases the TP53 mutation rate.	*APC* hypermethylation was associated with p53 mutation in 208 CRC.	Suehiro et al., 2008; Stamos and Weis, 2013 [43,44]
*MGMT*	O^6^-methylguanine-DNA methyltransferase	MGMT downregulation by promoter hypermethylation predisposes to p53 mutations. *MGMT* hypermethylation is associated with G > A mutations in *TP53.*	* MGMT * promoter hypermethylation was significantly correlated to G:C > A:T transition p53 mutations in 314 CRC.	Esteller et al., 2001a [45]
			*MGMT* methylation was associated with G > A mutations in the TP53 gene.	Deng et al., 2008 [46]
			No association between *MGMT* methylation and *TP53* mutations in 261 CRC biopsies from Afro-American patients	Alonso et al., 2015 [47]
*PTX3*	Pentraxin 3	Tumor suppressor. PTX3 deficiency increases susceptibility to carcinogenesis by increasing DNA damage, p53 mutations, and inactivation of p53 downstream signaling (Mdm2, Bax, and Cdkn1a /p21).	Increasing *PTX3* promoter methylation from normal colon epithelium to adenomas and CRC.	Bonavita et al., 2015 [48]
*PCAF*	P300/CBP-associated factor	Trimethylated histone H3K27 binding in the *PCAF* promoter attenuated its transcription.	Decreased *PCAF* impairs the acetylation of p53 and attenuates the p53-dependent transcription of p21, which results in the increased cyclin D1 expression and Retinobla-stoma 1 phosphorylation as well as increased resistance to 5-fluorouracil.	Liu et al., 2019 [49]
*LACTB*	Lactamase β	Tumor suppressor. LACTB binds to the p53 C terminus to inhibit p53 degradation by MDM2.	LACTB was significantly downregulated in CRC due to promoter methylation and histone deacetylation.	Zeng et al., 2018 [50]
*TRIM67*	Tripartite motif containing 67	Tumor suppressor. Upon stress, p53 binds to *TRIM67* promoter and upregulates TRIM67 expression in a TRIM67/p53 self-amplifying loop. TRIMP67 interacts with the p53 C-terminus to inhibit p53 degradation by MDM2.	108/138 CRC (=79%) downregulated TRIMP67 expression due to promoter methylation. Demethylation by treatment with 5-aza-2′-deoxycytidine restored TRIM67 expression in CRC cells.	Wang et al., 2019 [51]
*COX2*	Cyclooxygenase 2	Role in CRC progression. Converts arachidonic acid to prostaglandins	Methylation in 12/93 (=13%) CRC and 7/50 (=14%) colorectal ademonas. COX2 methylation was inversely related to p53 mutations. Functional relationship unclear	Toyota et al., 2000a [52]
CIMP	CpG island methylator phenotype	High levels of DNA methylation may predispose to carcinogenesis.	*TP53* mutations in 10/41 (=24%) CIMP-positive CRC compared to 30/46 (=60%) CIMP-negative cases. Functional relationship unclear	Toyota et al., 2000b [53]
CIMP	CpG island methylator phenotype		CIMP correlated with wildtype p53. Functional relationship unclear	Konishi et al., 2007 [54]
miR-34a	Micro-RNA 34a	miR-34a acts as translational repressor.	miR-34a activates p53 by inhibiting its acetylation by MTA2 and HDAC1.	Kaller et al., 2011 [55]
			miR-34a methylation correlates with wild-type p53 in CRC and other tumor types.	Vogt et al., 2011 [56]
